# Structure and Dielectric Property of High-*k* ZrO_2_ Films Grown by Atomic Layer Deposition Using Tetrakis(Dimethylamido)Zirconium and Ozone

**DOI:** 10.1186/s11671-019-2989-8

**Published:** 2019-05-07

**Authors:** Junqing Liu, Junpeng Li, Jianzhuo Wu, Jiaming Sun

**Affiliations:** Research Center for Photonics and Electronics Materials, School of Materials Science and Engineering & National Institute for Advanced Materials, Nankai University, Tongyan Road 38, Tianjin, 300350 China

**Keywords:** Atomic layer deposition, ZrO_2_, Electrical property, Thermal annealing

## Abstract

High-*k* metal oxide films are vital for the future development of microelectronics technology. In this work, ZrO_2_ films were grown on silicon by atomic layer deposition (ALD) using tetrakis(dimethylamido)zirconium and ozone as precursors. The relatively constant deposition rate of 0.125 nm/cycle is obtained within the ALD temperature window of 200–250 °C. The film thickness can be precisely controlled by regulating the number of ALD cycle. The ZrO_2_ films formed at 200–250 °C have an O/Zr atomic ratio of 1.85–1.9 and a low content of carbon impurity. ZrO_2_ film begins to crystallize in ALD process above 210 °C, and the crystal structure is changed from cubic and orthorhombic phases to monoclinic and orthorhombic phases with increasing the deposition temperature to 350 °C. Moreover, the effect of annealing temperature on dielectric properties of ZrO_2_ film was studied utilizing ZrO_2_-based MIS device. The growth of the interface layer between ZrO_2_ and Si substrate leads to the decrease in the capacitance and the leakage current of dielectric layer in the MIS device after 1000 °C annealing. ZrO_2_ film exhibits the relatively high dielectric constant of 32.57 at 100 kHz and the low leakage current density of 3.3 × 10^−6^ A cm^−2^ at 1 MV/cm.

## Background

Gate dielectrics have been a continuous research interest due to their broad applications in nano- and microelectronics [1–3], such as metal oxide films for complementary metal oxide semiconductors (CMOS) [4] and dynamic random access memory (DRAM) [5]. However, the use of the traditional SiO_2_ comes to its limit due to the scaling of devices, hence it is urgent to develop next generation of gate dielectrics to replace SiO_2_ in semiconductor industry [6, 7]. Recently, alternative metal oxides have been extensively investigated, such as ZrO_2_, Ta_2_O_5_, HfO_2_, Nb_2_O_5,_ and TiO_2_. In particular, ZrO_2_ has been considered as an ideal candidate due to its relatively high dielectric constant and wide band gap, as well as excellent thermal and chemical stability [8, 9].

In the past decade, ZrO_2_ film has been successfully synthesized via vacuum-based vapor-deposition routes [8, 10–12] and sol-gel solution-deposition routes [13–15]. Among them, atomic layer deposition (ALD) has distinguished advantages over other routes based on saturated self-limiting surface reactions [16, 17], including thickness controllability, large-area uniformity, low deposition temperature, and structure conformality, which makes ALD play an important role in the fabrication of high-quality dielectric films [18, 19].

Several precursors have been successfully applied for deposition of ZrO_2_ films by ALD processes. Kukli et al. reported that ZrO_2_ films were grown from ZrI_4_ and H_2_O-H_2_O_2_ on *p*-Si(100) substrates using ALD technique. The relative permittivity measured at 10 kHz was 20–24 in the films deposited at 275–325 °C [20]. Kukli et al. reported that ZrO_2_ films were grown by ALD from ZrCl_4_ and H_2_O or a mixture of H_2_O and H_2_O_2_ on Si(100) substrates in the temperature range of 180–600 °C. The films grown at 180 °C contain 5–6 at.% of hydrogen and 4–5 at.% chlorine. The effective permittivity of ZrO_2_ films is 13–15, when the ZrO_2_ was deposited at 180–210 °C [21]. Putkonen et al. reported that ZrO_2_ films were deposited onto (100) silicon substrates by ALD using Cp_2_Zr(CH_3_)_2_ (Cp = cyclopentadienyl) and water as precursors at 200–500 °C. The effective permittivity was 12.5 for the 19.0-nm film. The leakage current density in a 1 MV/cm field was about 2.8 × 10^−5^ A/cm^2^ for the 19.0-nm ZrO_2_ film [22]. Lamperti et al. reported that ZrO_2_ films were deposited on Si (100) substrates by ALD at 300 °C from (MeCp)_2_ZrMe(OMe) as Zr precursor and using H_2_O or O_3_ as an oxygen source. The measured dielectric constant values after 800 °C annealing are *k*~ 24 and *k*~ 30 in the films [23]. Previously, the common precursors were ZrCl_4_ [8, 24] or ZrI_4_ [25, 26]. However, low reaction activities of halides lead to high deposition temperatures [27]. Moreover, the formation of corrosive by-products (e.g., HCl) can corrode the films and ALD systems. Recently, metal amides precursors have been regarded as the ideal replacements without corrosive halogen by-products. Moreover, they have much higher reactivity than metal halides, since the metal-nitrogen bond is significantly weaker than the metal-halide bonds. In particular, tetrakis(dimethylamido)zirconium (TDMAZr) has thermal stability [28], sufficient volatility [29], and high reaction activity in the vapor deposition process. Therefore, TDMAZr and H_2_O as reaction precursors have been used for ALD processes of ZrO_2_ films [30], Nevertheless, hydrogen and carbon impurities in ZrO_2_ films have severe effects on the electrical properties of thin films. The “dry” ALD process of metal precursor and ozone (O_3_) has attracted more and more interest, because O_3_ as oxidizing agent can avoid introducing hydrogen. Moreover, O_3_ can remove carbon impurity by forming volatile CO and CO_2_ [31]. In addition, O_3_ has been widely used in the fabrication of dielectric layer in order to achieve higher crystallinity accompanied by a higher dielectric constant.

In this work, ZrO_2_ films were deposited on silicon by ALD technology using TDMAZr as metal precursor and O_3_ as oxygen precursor. The dependences of growth rate, refractive index, crystal structure, and surface roughness on deposition temperature were investigated in details by optical ellipsometry, glancing angle incidence X-ray diffraction (GAXRD), X-ray photoelectron spectroscopy (XPS), and atomic force microscope (AFM). Post-deposition annealing was used to further improve the crystal quality of ZrO_2_ films. Most importantly, we evaluated the dielectric constant of ZrO_2_ film and researched the effect of annealing temperature on the dielectric properties of ZrO_2_ films by analyzing the capacitive behavior and leakage current of ZrO_2_-based MIS devices.

## Methods

ZrO_2_ films were grown onto oriented *n*-type silicon wafers using an ALD reactor (MNT Ltd.). High purity nitrogen gas (99.999%) was used as a carrying and purging gas. TDMAZr as metal precursor was heated to 70 °C and carried into a reactor by carrying gas flowing through the source bottle. O_3_ as oxidant precursor was generated from oxygen (99.999% purity) by an ozone generator (Newland Ltd.). The delivery lines were remained at 100 °C to keep the gas from condensing. TDMAZr and O_3_ entry alternately into the reaction chamber to conduct surface gas-solid chemical reactions. We need to ensure that O_3_ is sufficiently excessive (about 20,000 Pa) and the purging process is long enough. The ZrO_2_ thin films deposited on silicon were annealed for 2 h under nitrogen atmosphere.

The thickness and refractive index of all samples were measured by an ellipsometer. The crystal structure of ZrO_2_ film was analyzed by glancing angle incidence X-ray diffraction (GAXRD). The chemical component and chemical state of ZrO_2_ film were analyzed by X-ray photoelectron spectroscopy (XPS, Sigma Probe, ThermoVG) using a monochromatic Al Ka source (1486.7 eV) to excite the photoelectrons. The positions of all the peaks were calibrated with the C 1s peak assigned at 284.6 eV. The surface morphology and the root-mean-squared (RMS) roughness of ZrO_2_ film were examined with an atomic force microscope (AFM, JSPM-5200, JEOL Co.). Current-voltage (I–V) measurement was carried out by a Keithley 2410 1100 V source measurement unit (Keithley Instruments Inc., Cleveland, OH, USA) and capacitance-voltage (C-V) measurement was carried out by TH2828S LCR meter (TONGHUI ELECTRONICS). All the measurements were completed at room temperature.

## Results and Discussion

**Fig. 1:**
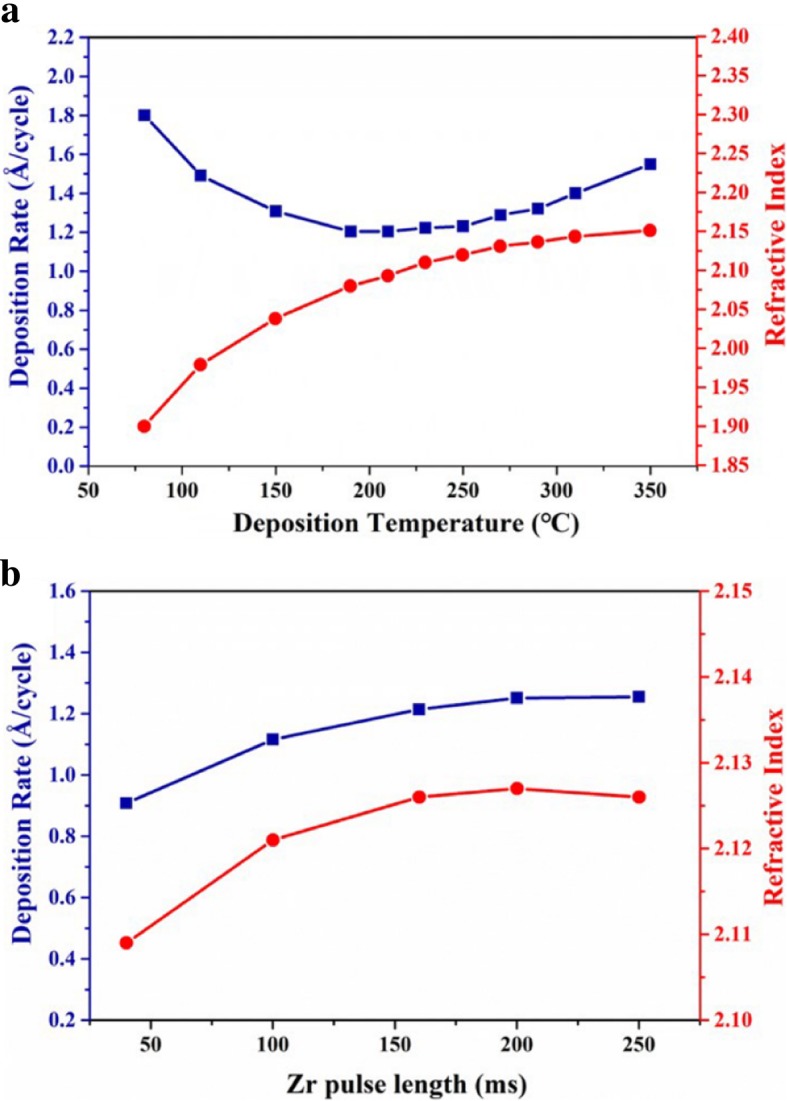
The changes of deposition rate and refractive index for ZrO_2_ thin films as a function of deposition temperature (**a**) and Zr precursor pulse time (**b**)

Deposition temperature plays a critical role in ALD technology. Figure 1a illustrates the dependences of the deposition rate and refractive index on the deposition temperature. The deposition rate constantly increases with decreasing the deposition temperature below 200 °C, which is due to the physical adsorption between the silicon substrate and the reaction precursors at the low deposition temperatures. From the profiles of refractive index as a function of deposition temperature, the constant decrease of refractive index below 200 °C means that thin films become looser, which also verifies that there is strong physical adsorption during the films’ growth at the low deposition temperatures. The deposition rate of 1.25 Å/cycle can be considered as quite a stable value in the narrow temperature range of 200~250 °C, in which the chemical adsorption plays a dominant role based on saturated self-limiting surface reaction. Then the deposition rate obviously increases with increasing the deposition temperature above 250 °C. These results can be attributed to the thermal decomposition of TDMAZr at the high deposition temperatures, which leads to CVD-like deposition. Figure 1b shows the dependences of the deposition rate and refractive index on the pulse time of TDMAZr. The growth rate at about 1.25 Å/cycle is observed when pulse time is more than 200 ms, which verifies the self-limiting film growth.

**Fig. 2:**
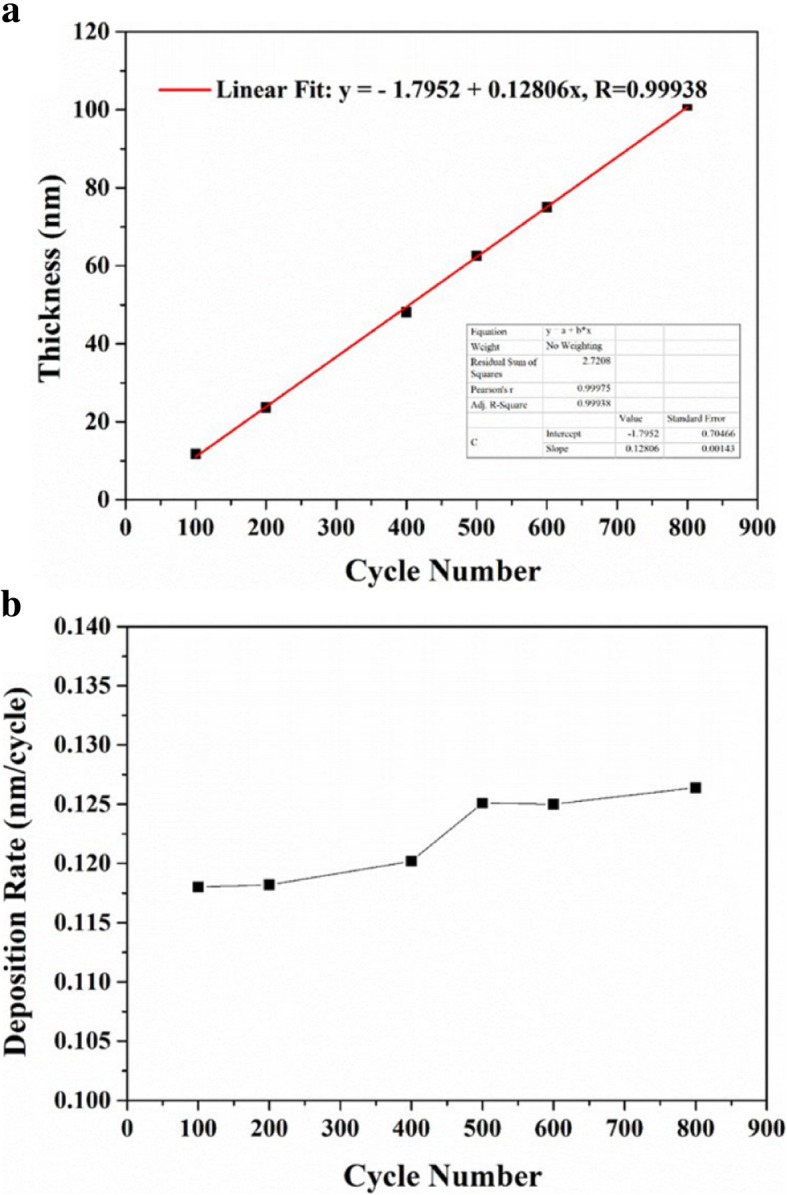
The changes of thickness (**a**) and deposition rate (**b**) for ZrO_2_ thin films as a function of the number of deposition cycles. The deposition temperature is 250 °C

Figure 2a depicts the dependence of thickness on the number of ALD cycles in ALD temperature window. The thickness of ZrO_2_ films on silicon shows a linear relationship with the number of ALD cycles, and the fitted formula is *y =* − 1.7952 + 0.12806x, *R =* 0.99938, which demonstrates that the thickness of thin the film can be precisely controlled by regulating the number of ALD cycles in ALD temperature window. Figure 2b shows the dependence of deposition rate on the number of ALD cycles. The deposition rate slightly increases with increasing the number of deposition cycles. Relatively low deposition rate at the beginning of film deposition can be attributed to crystal lattice mismatch and slow nucleation on silicon.

**Fig. 3:**
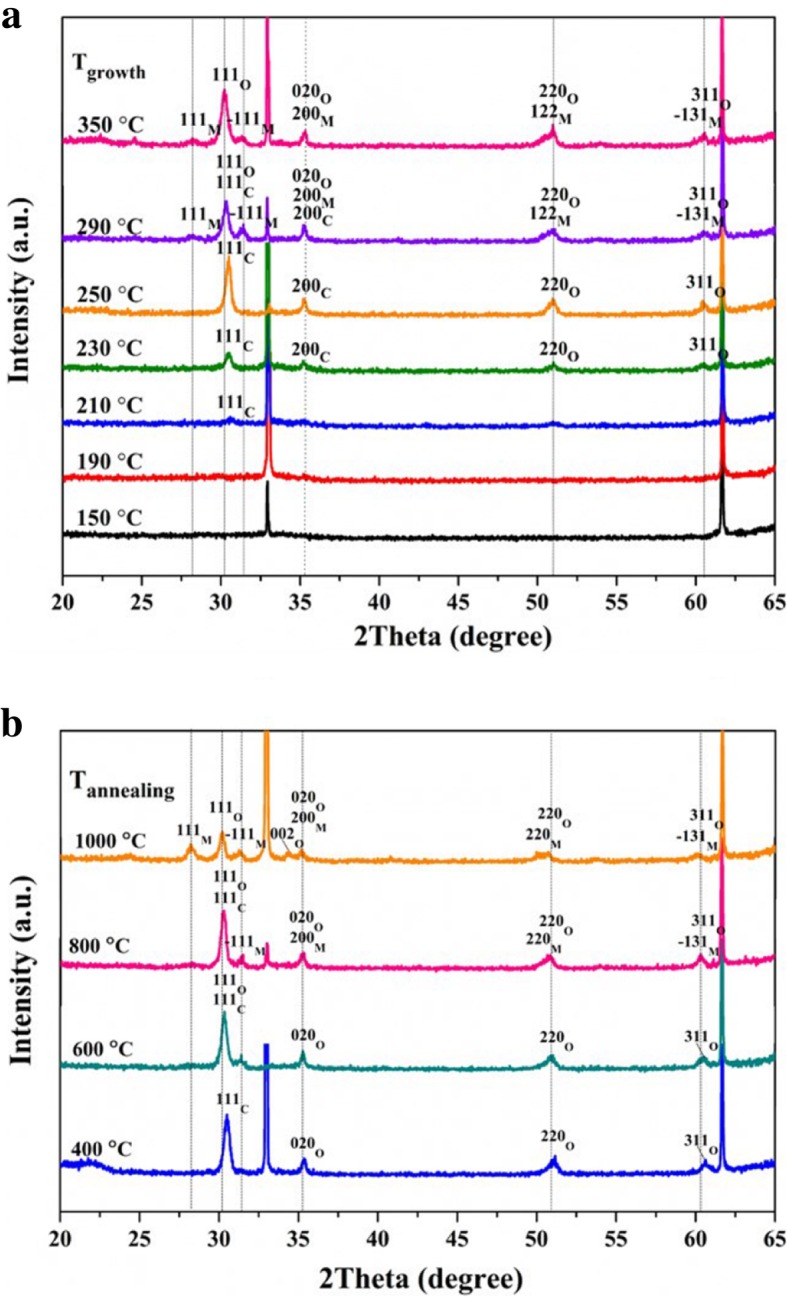
The GAXRD patterns for (**a**) ZrO_2_ films deposited at 150~350°C and (**b**) ZrO_2_ films annealed at 400~1000 ℃ (deposited at 250°C)

The crystallographic structure of the ZrO_2_ thin films deposited at 150~350 °C was detected by GAXRD. As shown in Fig. 3a, ZrO_2_ films begin to crystallize at the deposition temperature of 210 °C. The reflections at 30.5° and 35.3° are indexed to the (111) and (200) lattice planes of cubic phase (PDF Card 027-0997), respectively. The reflections at 51° and 60.5° are indexed to the (220) and (311) lattice planes of orthorhombic phase (PDF Card 037-1413). With increasing the deposition temperature to 290 °C, the (111), (− 111), (200), (122) and (− 131) lattice planes of stable monoclinic phase begin to appear at 28.3°, 31.4°, 35.3°, 50.6°, and 60° (PDF card 007-0343), respectively [24]. Moreover, the reflections at 30.5° and 35.3° begin to slightly shift, which indicates the phase change from cubic phases to orthorhombic phase. When the deposition temperature reaches 350 °C, the lattice planes of the cubic phase disappear, and the dominant crystallographic phases in ZrO_2_ films are both orthorhombic and monoclinic phase. Post-deposition annealing is regarded as a necessary process to eliminate defects and improve the crystal quality. Figure 3b showed the GAXRD patterns of the ZrO_2_ films (deposited at 250 °C) annealed at 400~1000 °C. It can be found that main crystal structures are both cubic and orthorhombic phases in the ZrO_2_ film annealed at 400 °C. However, monoclinic phase begins to appear, and the intensities of reflections at 28.3° and 31.4° significantly increase with increasing annealing temperature to 1000 °C. Meanwhile, the phase change from cubic phases to orthorhombic phase can be seen from the shift of the reflection for (111) plane. From the above analysis, both deposition temperature and annealing temperature have significant effects on the crystallographic structure of ZrO_2_ film.

**Fig. 4:**
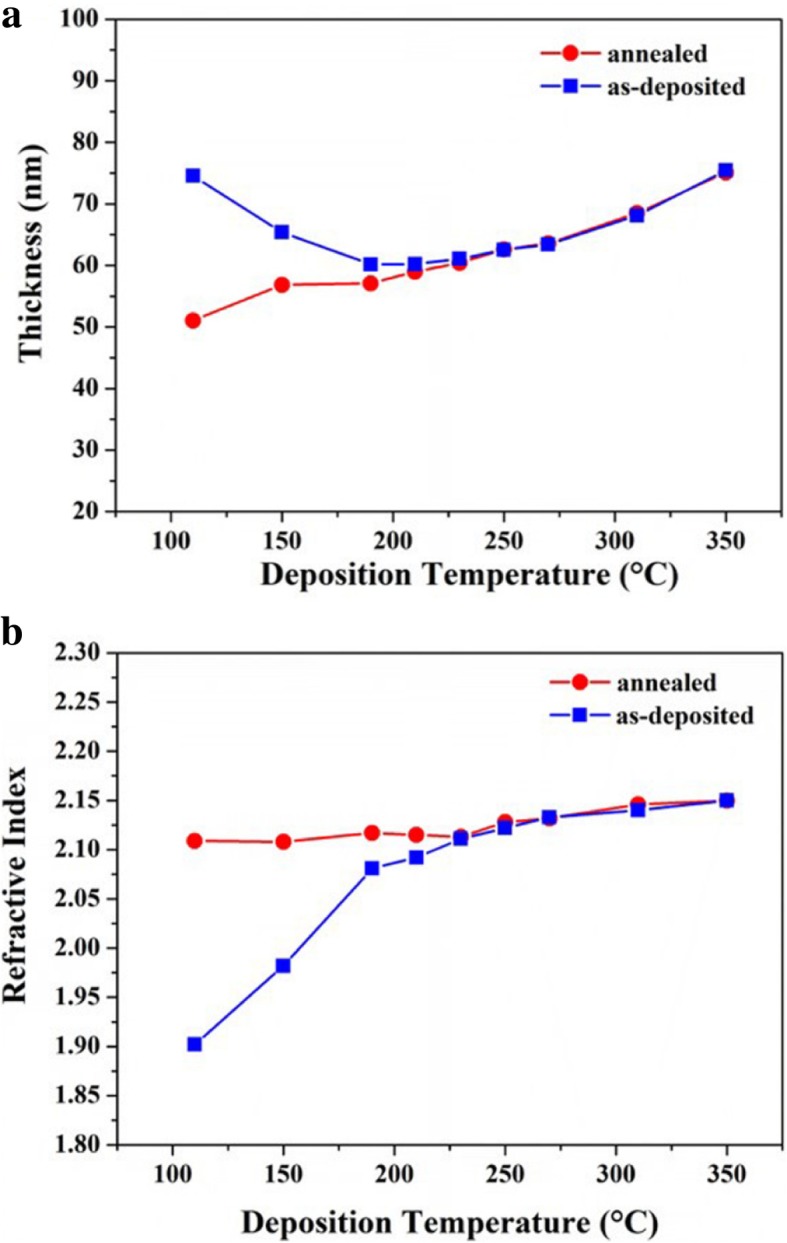
The changes of thickness (**a**) and refractive index (**b**) for the films deposited at 110~350 °C after post-deposition annealing at 400 °C

Figure 4 shows the changes of thickness and refractive index for the films deposited from 110 to 350 °C after 400 °C annealing. The thickness of the ZrO_2_ film deposited below 210 °C significantly decreases after 400 °C annealing, meanwhile, the refractive index correspondingly increases, which demonstrates the thin film becomes denser after post-deposition annealing. However, the thickness and refractive index have no change for the thin film deposited above 210 °C after annealing at 400 °C, which may be because the crystal growth of thin films have been sufficient, namely, the sizes of grains are no longer obviously increase above 210 °C.

**Fig. 5:**
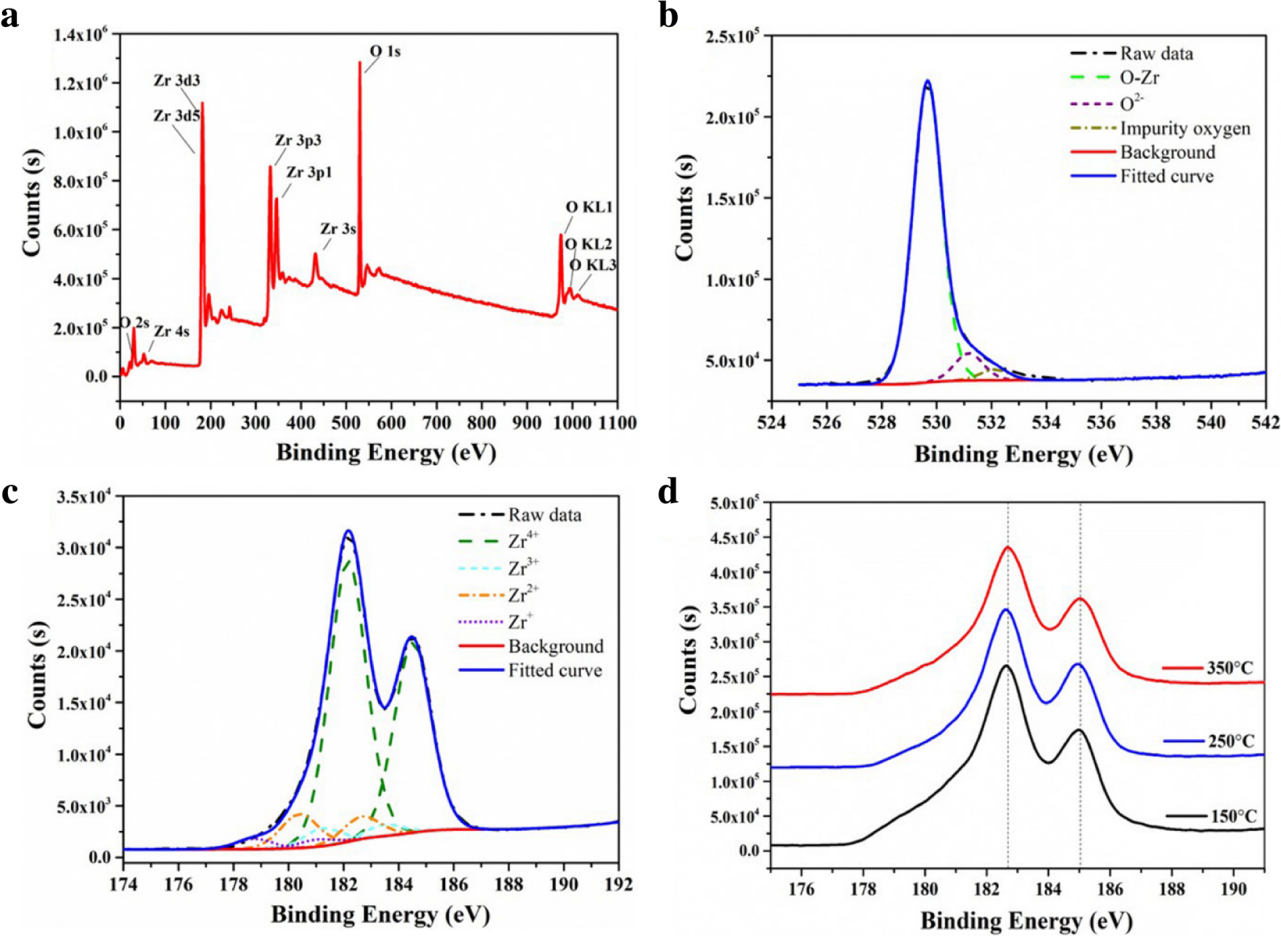
The full spectrum scan of XPS (**a**), the fine spectra of O 1 s (**b**), and Zr 3d (**c**) for ZrO_2_ film deposited on Si(100) at 250 °C, and the fine spectra of Zr 3d for ZrO_2_ films deposited at 150, 250, and 350 °C (**d**)

The chemical component and chemical state of ZrO_2_ thin film on silicon were carefully investigated by XPS. The thickness of ZrO_2_ film in XPS measurement is about 50 nm. Moreover, surface cleaning was carried out by etching before XPS measurement. As described in Fig. 5a, a full spectrum result shows the existences of oxygen and zirconium in ZrO_2_ thin film deposited at 250 °C, with no obvious impurity peak, which confirms the high purity of ZrO_2_ thin film. Moreover, the ratio of O/Zr in ZrO_2_ films deposited at 200~250 °C is 1.85–1.9, which is less than the stoichiometric ratio of ZrO_2_ due to the formation of oxygen vacancy in the ZrO_2_ film. An asymmetric O 1s XPS spectrum was obtained in this study, shown in Fig. 5b. The O 1s high-resolution spectrum is composed of two overlapping components, with a lower binding energy peak (529.7 eV) and the higher binding energy (531.3 eV), which result from the Zr–O of oxides and O–C of by-product, respectively. In Fig. 5c, the high-resolution spectrum of Zr 3d shows two peaks at 182.4 eV and 184.7 eV, which corresponds with the features of Zr 3d_5/2_ and 3d_3/2_, respectively. It should be noted that shoulders of the Zr 3d_5/2_ peak appear on the lower binding energy side. The Zr 3d spectra are decomposed satisfactorily in terms of four different oxidation states (Zr^4+^~Zr^+^) of Zr equally spaced in 1.06 eV (one-fourth of the chemical shift between Zr^0^ and Zr^4+^) per oxidation state with respect to the metal [32–34]. All the oxidation states are observed in all the films prepared, and the Zr^4+^ state is the major component in the films due to the effect of suboxide species. In addition, deposition temperature just has little impact on the binding energy of Zr 3d and the shape of the peaks in spectra, which demonstrates that the deposition temperatures within the range of 150~350 °C cannot change the chemical state of Zr 3d.

**Fig. 6:**
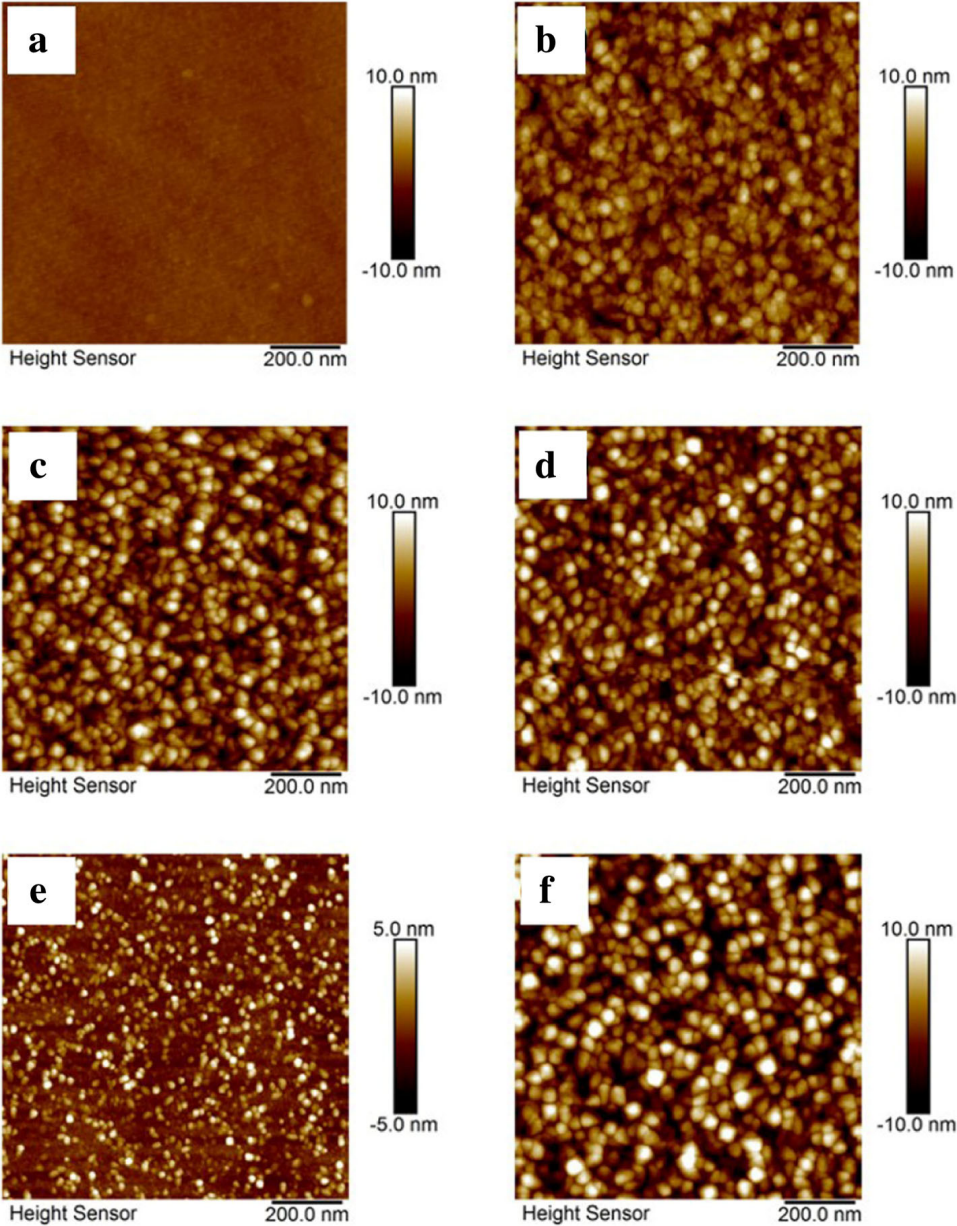
AFM surface plots of ZrO_2_ films deposited at 150 °C (**a**) and 350 °C (**b**), ZrO_2_ films (deposited at 250 °C) annealed at 400 °C (**c**) and 1000 °C (**d**), and ZrO_2_ films (deposited at 250 °C) with 200 ALD cycles (**e**) and 800 ALD cycles (**f**)

Roughness and morphology were studied by AFM. Figure 6 displays representative AFM images of ZrO_2_ thin films, which illustrates that the surfaces of ZrO_2_ thin films are uniform and smooth. As shown in Fig. 6a and b, the root-mean-square (RMS) roughness for the ZrO_2_ films deposited at 150 °C and 350 °C are 0.293 nm and 1.718 nm, which are 0.448% and 2.277% of the films aggregate thicknesses, respectively. By comparing, the result suggests that amorphous ZrO_2_ films are much smoother than crystalline film. Although the RMS roughness does not provide direct evidence of the detailed evolution of crystallographic phases in oxide thin films, the increase in surface roughness could be generally ascribed to the formation and growth of the grain in the thin film. As shown in Fig. 6c and d, ZrO_2_ films deposited at 250 °C were annealed at 400 °C and 1000 °C, respectively. The RMS roughness has no significant change with the increasing annealing temperature, which indicates that 400 °C annealing has led to sufficient crystal growth and there is no obvious increase in grain size. As shown in Fig. 6e and f, the thickness of ZrO_2_ film controlled by ALD cycles have an obvious effect on the RMS roughness of the thin film. By comparing, the RMS roughness increases with the increasing number of ALD cycles, which can be explained by that the crystal growth is limited when the thickness of the film is very thin.

**Fig. 7:**
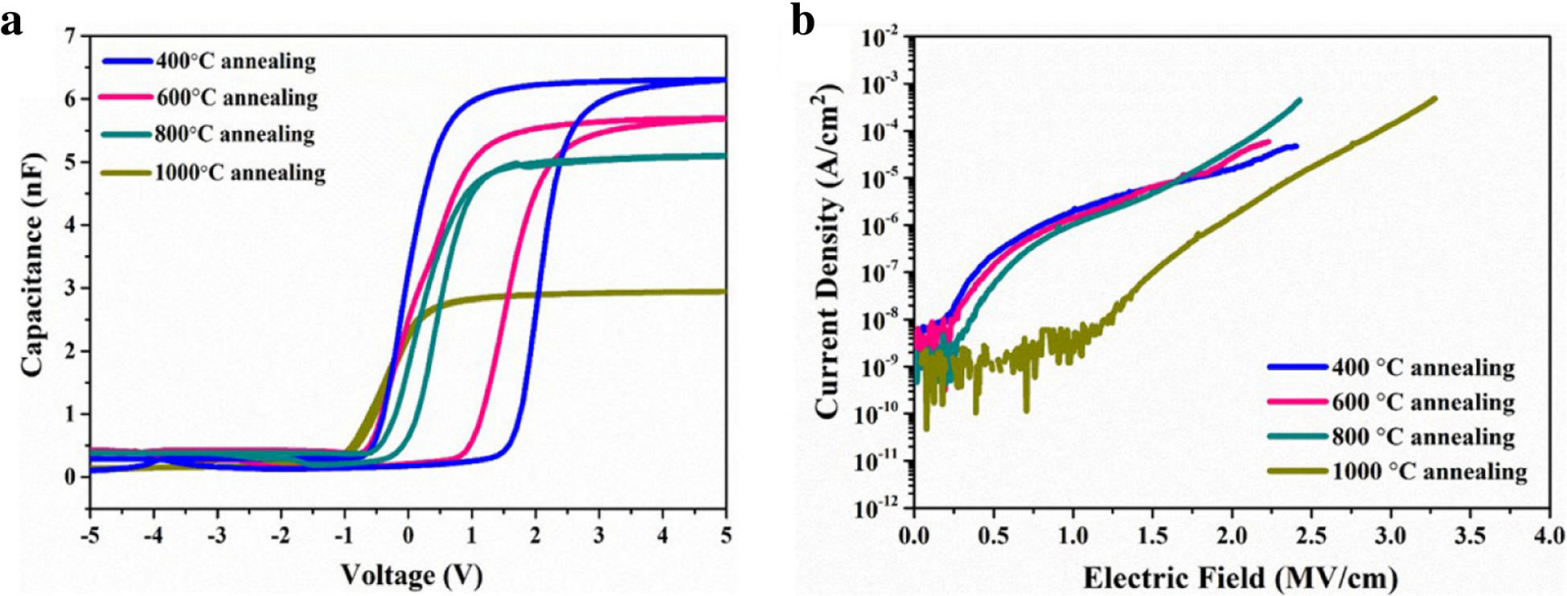
The C-V (**a**) and J-E curves (**b**) for ZrO_2_-based MIS devices (with 500 cycles) as a function of annealing temperature

**Fig. 8:**
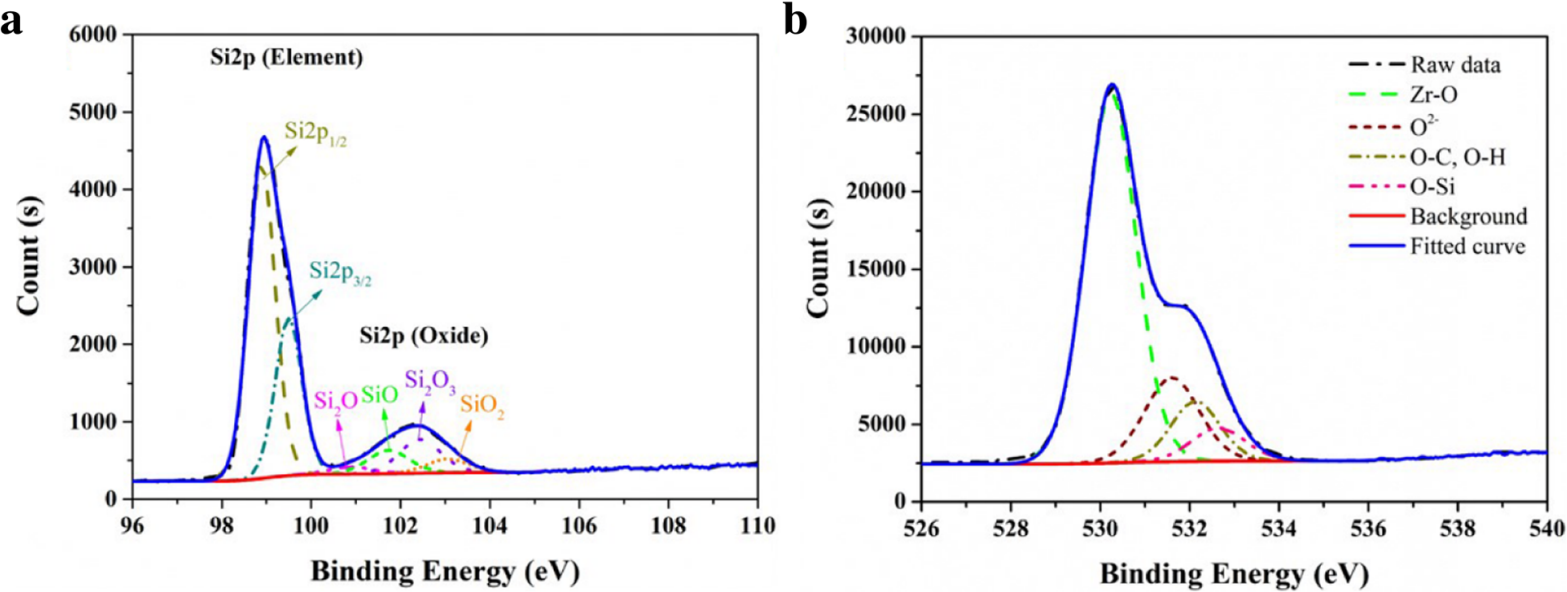
The fine spectra of Si 2p and O 2s for the ZrO_2_ film deposited at 250

Capacitance-voltage (C-V) and current density-electric field (J-E) measurements were conducted to assess the electrical properties of MIS devices with ZrO_2_ films as dielectric layers with various annealing temperatures. As shown in Fig. 7a, the capacitance constantly decreases with increasing annealing temperature. Moreover, obvious hysteresis effects between the forward and backward sweep (clockwise) were observed in the samples annealed at 400 °C and 600 °C, and then the hysteresis effect decreases or even disappears with increasing annealing temperature to 1000 °C. The hysteresis effect should be attributed to the charge trap/detrap in the interface between the ZrO_2_ film and Si substrate [35–37]. The oxygen in ZrO_2_ film diffuses toward the interface to result in the further growth of SiO_x_ layer between ZrO_2_ and Si substrate with increasing the annealing temperature during post-deposition annealing [37–39]. To prove the presence of SiO_x_ interface layer, ultrathin ZrO_2_ (2.5~3 nm) deposited on Si substrate was used to XPS measurement. As shown in Fig. 8a, in Si 2p spectrum, the signal from the Si substrate (98.81 eV and 99.45 eV) could be clearly observed due to the thinness of the films, and the signal from interface layer (100.38 eV, 100.88 eV, 102.44 eV, and 103.05 eV) indicates the existence of Si sub-oxides. As shown in Fig. 8b, in the O 1s spectrum, the binding energy of 532.72 eV is identified as Si–O in SiO_*x*_. These results can prove the formation of SiO_*X*_ interface layer. The SiO_x_ layer can reduce interface defect state density and improve the interface quality [39]. Therefore, the increase in annealing temperature can reduce the hysteresis effect. Figure 7b depicts J-E curves for ZrO_2_-based MIS devices. With increasing the annealing temperatures from 400 °C to 800 °C, there is no significant reduction in current densities. However, with increasing the annealing temperature to 1000 °C, the leakage current density decreases to 2.85 × 10^−9^ A cm^−2^ at 1 MV cm^−1^. It can be attributed to the further growth of SiO_x_ intermediate layer with wide band gap during 1000 °C annealing.

**Fig. 9:**
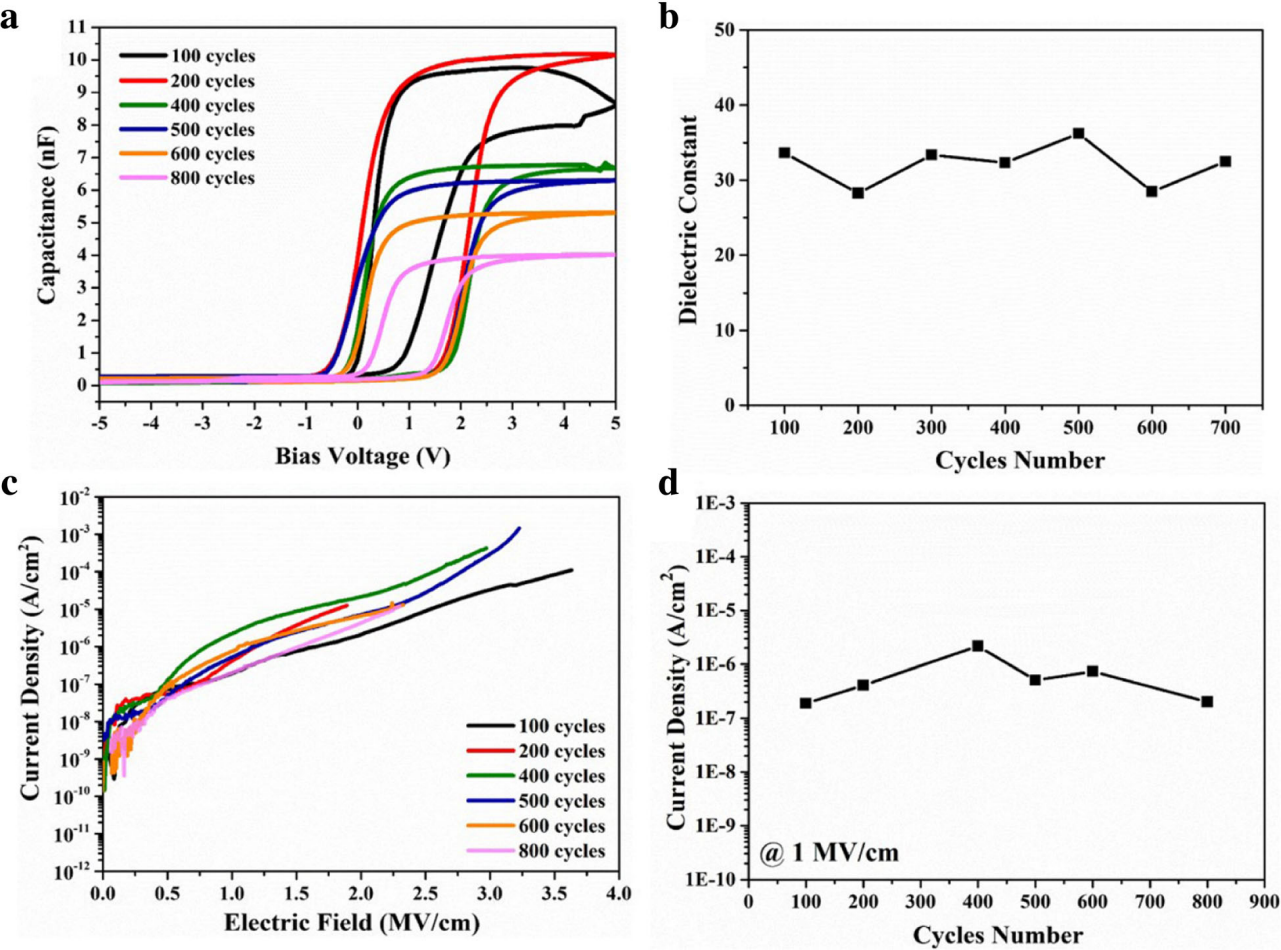
The C-V (**a**) and J-E curves (**b**) for ZrO_2_-based MIS devices with 100~800 cycles, the changes of dielectric constant (**c**), and the current density at 1 MV/cm (**d**) as a function of the number of deposition cycle

**Fig. 10:**
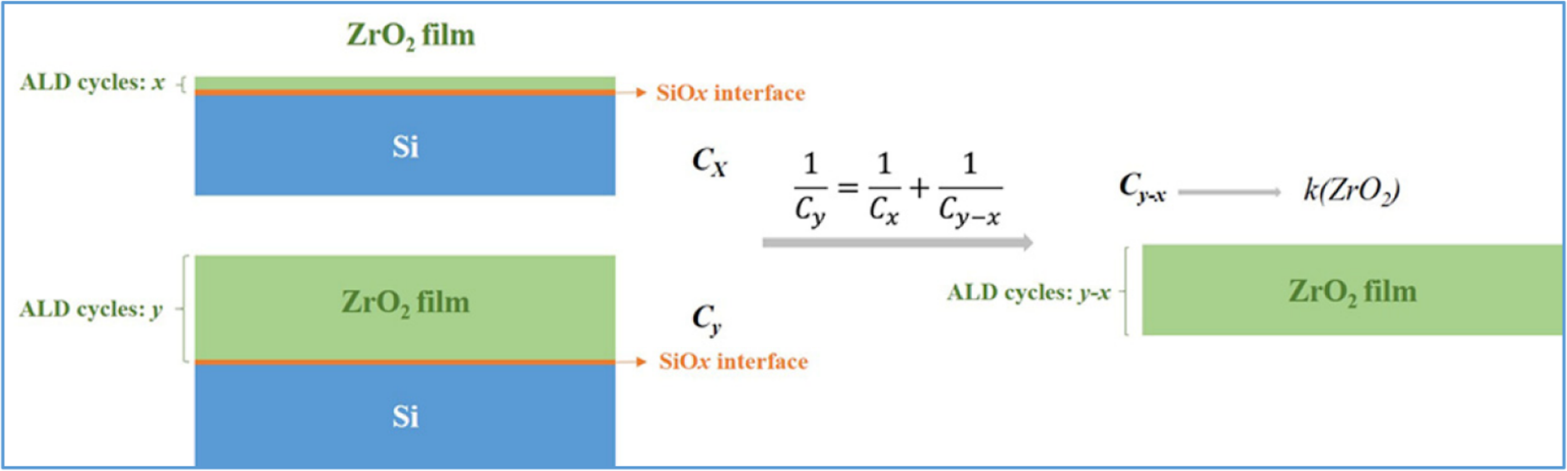
Schematic diagram of dielectric constant calculation

**Fig. 11:**
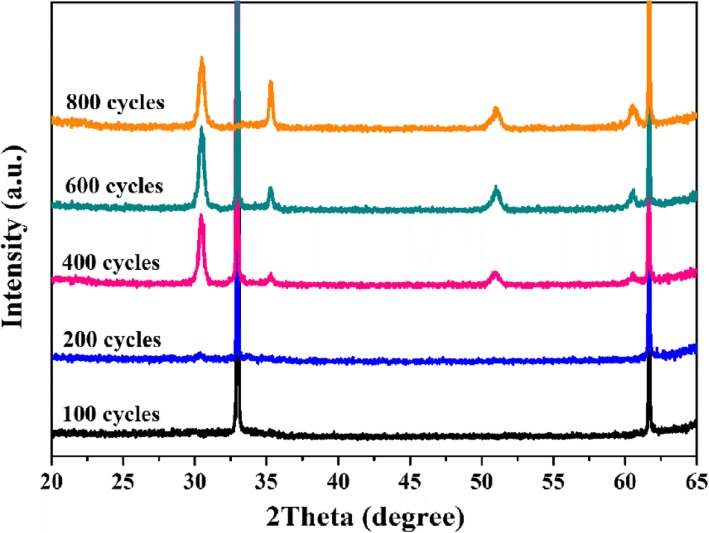
The GAXRD patterns for the ZrO_2_ films deposited at 250 °C with various ALD cycles

Figure 9a shows the effect of ZrO_2_ thickness on the capacitance behavior of ZrO_2_-based MIS devices. The capacitance constantly decrease with increasing ALD cycles (or thicknesses), which is attributed to the increase in the physical thickness for ZrO_2_ dielectric layer. To evaluate the dielectric constant of the ZrO_2_ dielectric layer, the effect of interfacial SiO_*x*_ layer was eliminated in calculation, as shown in Fig. 10. A similar dielectric constant for ZrO_2_ films with various thicknesses was obtained in Fig. 9b. The average value of the dielectric constant of ZrO_2_ film is 32.57. These results indicate that the film quality is reliable for ZrO_2_ films with various thickness deposited by ALD. Figure 9c and d illustrate that the increase in the physical thickness for ZrO_2_ films with 400–800 ALD cycles can decrease leakage current densities. However, it should be noted that the leakage current density remains low level when the cycle number are 100 and 200, which is due to less leakage paths in the thin film with limited crystallization. As shown in Fig. 11, the GAXRD patterns for the ZrO_2_ films with various ALD cycles (or thickness) illustrate the crystallization is inhibited in ZrO_2_ thin film with 100 and 200 ALD cycles.

## Conclusions

High-quality ZrO_2_ thin films were deposited on *n*-type silicon wafers by ALD technology using tetrakis(dimethylamido)zirconium and ozone as precursors. ALD temperature window for ZrO_2_ film is 200–250 °C, and the deposition rate is a relatively constant at 0.125 nm/cycle. The thickness of the thin film can be precisely controlled by regulating the number of ALD cycles. The ZrO_2_ films formed at 200~250 °C have an O/Zr atomic ratio of 1.85–1.9 and a low content of carbon impurity. ZrO_2_ film begins to crystallize in the ALD process above 210 °C, and the crystal structure is changed from cubic and orthorhombic phases to monoclinic and orthorhombic phases with increasing the deposition temperature to 350 °C. Moreover, a similar phase transition also can occur with increasing the annealing temperature from 400 to 1000 °C. The effect of annealing temperature on dielectric properties of ZrO_2_ film was studied utilizing ZrO_2_-based MIS device. The growth of the interface layer between ZrO_2_ and Si substrate leads to the decrease in the capacitance and the leakage current of dielectric layer in the MIS device after 1000 °C annealing. ZrO_2_ film exhibits the relatively high dielectric constant of 32.57 at 100 kHz and the low leakage current density of 3.3 × 10^−6^ A cm^−2^ at 1 MV/cm.

## Abbreviations

AFM: Atomic force microscope
ALD: Atomic layer deposition
CMOS: Complementary metal oxide semiconductors
C-V: Capacitance-voltage
DRAM: Dynamic random access memory
I-V: Current-voltage
O_3_: Ozone
RMS: Root-mean-squared
XPS: X-ray photoelectron spectroscopy
XRD: X-ray diffraction
Zr[N(CH_3_)_2_]_4_: Tetrakis(dimethylamido)zirconium
